# Mapping Forest Structure Using UAS inside Flight Capabilities

**DOI:** 10.3390/s18072245

**Published:** 2018-07-12

**Authors:** Karel Kuželka, Peter Surový

**Affiliations:** Faculty of Forestry and Wood Sciences, Czech University of Life Sciences Prague, 16500 Praha, Czech Republic; surovy@fld.czu.cz

**Keywords:** unmanned aerial system (UAS), vision positioning system, obstacle sensing, photogrammetry, point cloud, forestry, diameter at breast height (DBH)

## Abstract

We evaluated two unmanned aerial systems (UASs), namely the DJI Phantom 4 Pro and DJI Mavic Pro, for 3D forest structure mapping of the forest stand interior with the use of close-range photogrammetry techniques. Assisted flights were performed within two research plots established in mature pure Norway spruce (*Picea abies* (L.) H. Karst.) and European beech (*Fagus sylvatica* L.) forest stands. Geotagged images were used to produce georeferenced 3D point clouds representing tree stem surfaces. With a flight height of 8 m above the ground, the stems were precisely modeled up to a height of 10 m, which represents a considerably larger portion of the stem when compared with terrestrial close-range photogrammetry. Accuracy of the point clouds was evaluated by comparing field-measured tree diameters at breast height (DBH) with diameter estimates derived from the point cloud using four different fitting methods, including the bounding circle, convex hull, least squares circle, and least squares ellipse methods. The accuracy of DBH estimation varied with the UAS model and the diameter fitting method utilized. With the Phantom 4 Pro and the least squares ellipse method to estimate diameter, the mean error of diameter estimates was −1.17 cm (−3.14%) and 0.27 cm (0.69%) for spruce and beech stands, respectively.

## 1. Introduction

Sustainable forestry that provides important ecosystem services [1] requires careful planning that is predominantly based on precise inventory data. The increasing costs of human labor in developed countries has encouraged forest owners to increase the efficiency of data collection and processing. Because traditional forest inventory methods demand large investments of time and resources, alternative methods to help simplify the assessment of defining parameters of forest trees and stands are being developed [2,3,4,5]. In recent years, special attention has been given to non-contact measurement methods based on advanced technology and recent computer vision techniques that provide precise three-dimensional (3D) data that allow for the reconstruction of forest stands and estimates of their parameters. The novel methods are based on the improving technologies of laser scanning and multi-view stereophotogrammetry.

Laser scanning methods utilize light detection and ranging (LiDAR) technology for precise range measurement of surrounding objects; they can provide 3D positions of up to one million points per second with millimeter-level precision. Extensive detailed information about tree and forest stand parameters can be derived from data obtained by a terrestrial laser scanner (TLS). With TLS 3D point clouds, we can evaluate virtually any dimension of the objects, including basic mensurational parameters of trees, such as diameter at breast height (DBH) and height. Stem diameters can be estimated by fitting circles or other plane figures to 2D cross-section perimeter points [6] or by fitting cylinders to 3D point clouds of stem sections [7]; the accuracy of DBH estimations currently ranges from one to several centimeters depending on the fitting method. Tree heights are more difficult to estimate because the laser beams rarely hit the real top of the tree; height estimation errors range from 0.8 m to several meters [8]. Stem curvature and detailed tree architecture can also be assessed [3,4,8]. The automatic detection and mapping of forest trees is also possible using TLS point clouds [9] and they can provide precise data for accurate, non-destructive estimates of aboveground biomass [10] and changes in aboveground biomass over time [11]. Because of its millimeter-level accuracy and the wide spectrum of possible applications, TLS is expected to be used operationally in forest inventories as soon as best practices are identified and appropriate data processing techniques become available [8,12]. However, the current price of laser scanning devices is rather prohibitive, with some costing as much as $100,000 USD, thus TLS systems are not commonly used for forest inventories [2]. Standard aerial laser scanning methods do not allow for the reconstruction of individual stems in forest stands due to their low density point clouds, although some recent studies [3,7] have used a laser scanner deployed on an unmanned aerial system (UAS) to estimate individual tree diameters. However, dense canopies often mask tree stems and the success rate of tree detection with this approach is limited [7]. An unmanned ground vehicle equipped with LiDAR has also been successfully used to map a forest stand [13], but further testing in alternative terrains and forest types is needed.

The multi-view stereophotogrammetry approach is a low-cost alternative to LIDAR that can also produce a 3D point cloud. This approach, which simply requires a commercial off-the-shelf digital camera, is a recent development triggered by advances in computer vision, such as the scale-invariant feature transform (SIFT) [14] and speeded up robust features (SURF) [15] algorithms. It can produce reconstructed 3D surfaces using image sequences acquired with digital cameras [16]. The Structure from Motion (SfM) technique utilizes a set of features that are identified in subsequent images to calculate camera positions in a local 3D coordinate system. Subsequently positions of the identified features are derived; resulting in a 3D point cloud representing surfaces of reconstructed objects. With a sufficient number of overlapping images, the positions of individual points that represent the surface of forest trees can reach sub-centimeter accuracy [17]. Because of the different processes of origin, the nature of LIDAR and photogrammetry point clouds and their ability to reconstruct vegetation structure also differ. While photogrammetry point clouds exhibit more detail about the outer surfaces of vegetation and canopy [18], they lack the ability of laser beams to penetrate the canopy and record greater detail in the structure of forest stands [19].

The potential of the SfM technique with the use of a low-cost hand-held camera to reconstruct tree structure and produce estimates of tree metrics was shown by [20]. The SfM technique has also been successfully utilized in general vegetation 3D mapping [21,22], tree detection and positioning [1,23], forest biomass prediction [24], and forest tree stem reconstructions [25,26], among others.

Photogrammetric 3D forest stand models have recently been developed using either tree stem surface reconstructions derived from imagery acquired in terrestrial surveys [1,26], or canopy reconstructions derived from aerial surveys above the forest stands, mostly utilizing UASs [18,27]. Direct stem measurements using aerial photogrammetric data require clear visibility of stems from multiple directions, and this is usually difficult. The latest generation of small commercial UASs are equipped with high-precision vision positioning systems and obstacle sensing and avoidance systems, which allows the UASs to operate safely inside forest stands and collect detailed image data that can be used to reconstruct the interior structure of forest stands. This study reports the possibilities of utilization of inside flight capabilities for forest structure mapping.

## 2. Research Area

**Figure 1:**
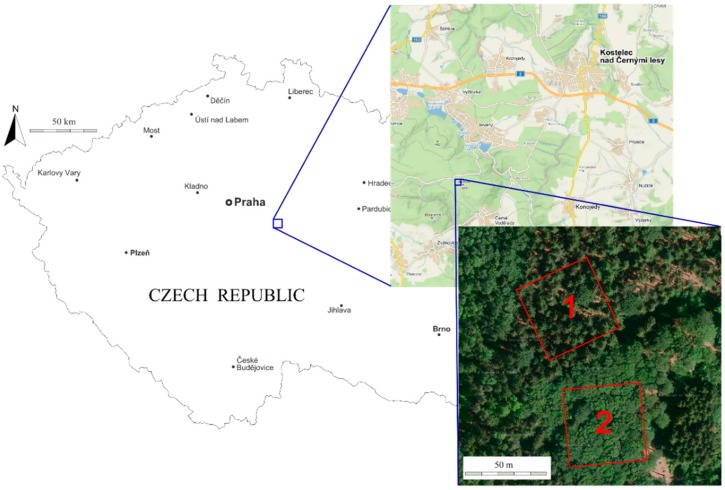
Location of spruce (**1**) and beech (**2**) research plots (Adapted from http://d-maps.com).

The research area is situated in the Central Bohemian Region of the Czech Republic at the School Forest Enterprise of the Czech University of Life Sciences, Prague Lat: 49.956 N, 14.794 E (Figure 1). The altitude is 500 m above mean sea level and the topography is generally flat.

**Figure 2:**
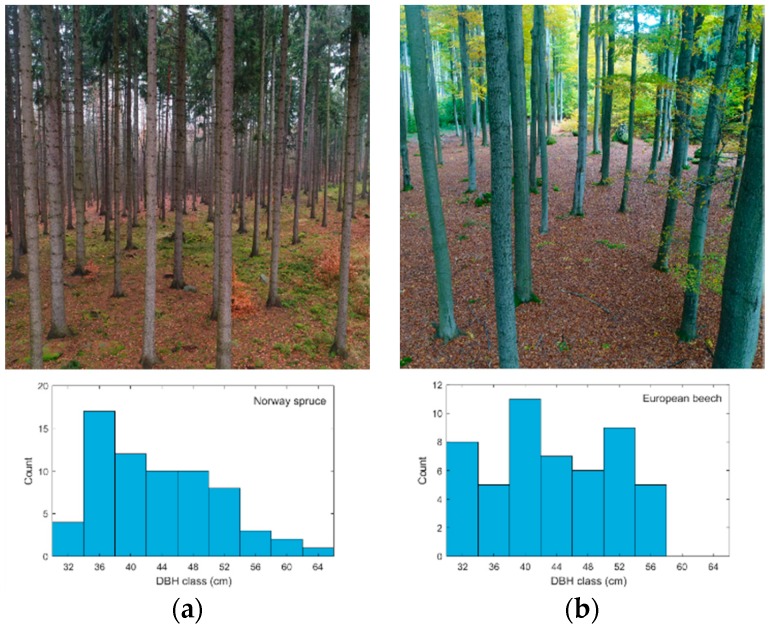
The real forest structure of the spruce (**a**) and beech (**b**) research plots. Photos illustrate the forest stands, and histograms show the real distribution of diameters at breast height (DBH) of trees based on field survey.

We selected the most abundant and economically most important representatives of coniferous and deciduous tree species of Central European forests, Norway spruce (*Picea abies* (L.) H. Karst.) and European beech (*Fagus sylvatica* L.), respectively. Two square research plots (50 m × 50 m) were established in two adjacent stands that were planted with different species, spruce and beech (Figure 2). The stands are homogenous mature even-aged stands with no understory and very little tree regeneration; see Table 1 for a detailed description of both forest stands. DBH histograms describe the diameter structure of trees in the research plots. In spruce research plot, most of the DBHs are between 32 and 52 cm, with a peak at 36 cm and a few larger trees reaching up to 64 cm. In beech plot, the DBHs range from 32 to 56 cm with the median point at about 42 cm.

## 3. Materials and Methods

### 3.1. Field Survey

Research plots were established using the TruPulse 360 laser rangefinder (Laser Technology, Inc., Centennial, CO, USA). Lengths of plot edges were directly measured using the rangefinder, and rectangularity of the plots was verified by diagonal measurements. Total station TOPCON GTS-210 (Topcon Corporation, Tokyo, Japan) was used to measure and record all of the tree positions, which we defined as the centers of the stem cross-sections at breast height (1.3 m from the base of the stem); positions were measured using the polar surveying method. Distances from the total station position were measured using a cube corner prism placed at the tree surfaces and angle offsets were applied; the position error did not exceed 5 cm. Diameter at breast height was measured using a diameter tape at a plane perpendicular to the axis of the stem at a height of 1.3 m from the base of the stem; diameters were recorded with a resolution of 1 mm. Tree heights were measured using a hypsometer VL5 Vertex Laser (Haglöf Sweden AB, Långsele, Sweden) based on the distance from the observer to the tree, measured using the laser finder, and vertical angle from the top of the stem to the base of the stem using the clinometer; heights were recorded with a resolution of 0.1 m.

### 3.2. Image Acquisition

We used two small commercially available UASs, DJI Phantom 4 Pro and DJI Mavic Pro (Dá-Jiāng Innovations Science and Technology Co. Ltd., Shenzhen, China), for image acquisition. Both models were chosen because of their ability to provide geotagged images. Another advantage of both models was the systems for obstacle sensing and avoidance, which increase flight safety on the interior of the forest stand. Both models were equipped with a high-precision vision positioning system, thus allowing them to precisely geotag images in conditions with a poor global navigation satellite system (GNSS) signal under forest canopies. Precise geotagging of the images is crucial for accurate scaling and georeferencing of the associated 3D point cloud. The main difference between the two models is the technical parameters of RGB sensors, which may result in differences in their applicability for 3D photogrammetry mapping. Based on differences in their specifications (Table 2), especially sensor size, the camera of the Phantom 4 Pro is supposed to acquire images with more detail and less noise. Moreover, its mechanical shutter does not cause geometric distortion, which is typical during movement of the camera with an electronic shutter.

The flight trajectory followed a double zig-zag pattern, which consists of two zig-zag flights in perpendicular directions, similar to [26]. In each direction, the research plot was fully covered by 10 overflights (five forward and five backward) with an average spacing between trajectory segments of 5 m. The flight height was 8 m above the ground surface during the entirety of the flight. The final trajectory does not follow a regular pattern with straight flight lines because the flight trajectory had to conform to the irregular distribution of trees; this also meant that the flight could not be executed automatically according to a flight plan and the flight was piloted manually. The flight trajectory could not be exactly repeated for both UASs; therefore, also the flight duration, number of images and image positions differ.

Images were acquired using integrated cameras of the UAVs in the flight direction with the vertical angle −45° from the horizontal plane. The cameras were set to a timed shot interval of 2 s for the Phantom 4 Pro and 3 s for the Mavic Pro; these are the highest frequencies available for each UAS. Images were recorded in a JPEG image format, and the Phantom 4 Pro and Mavic Pro were set with a maximum resolution of 20 MPix and 12 MPix, respectively, and exposure compensation was set to −1/3 EV.

### 3.3. Data Processing

Collected imagery and metadata were processed using Agisoft PhotoScan software [28]. We used the reference preselection of images and the high quality of alignment options to produce a 3D point cloud automatically georeferenced using the geotags embedded in images’ Exif metadata. The point cloud represents the ground surface and the surface of the lower parts of the tree stems below the crown, up to approximately 10 m above the ground.

### 3.4. Accuracy Assessment

We evaluated point cloud accuracy by comparing the DBHs of all trees in the plot derived from the point cloud with DBH acquired by field measurements. The points that represented the ground were automatically classified using Agisoft PhotoScan, and subsequent analyses were performed in MATLAB R2017b [29]. To separate point clusters that corresponded to individual trees, we conducted a cluster analysis using the hierarchical cluster tree method using the points that represented the surface of tree stems at breast height, which included all points at heights between 1.25 m and 1.35 m above the surface. Individual point clusters representing the perimeter of the stems were used to determine the DBH based on four automatic methods: (1) minimum bounding circle (CB), (2) convex hull (CH), (3) least squares circle (C), and (4) least squares ellipse (E).

The minimum bounding circle (CB) method defines the circle enclosing all points belonging to a specified cluster. Accuracy of the CB method can be negatively influenced by the irregularity of tree stems [2] and any associated noise in the point cloud; in both cases the error is supposed to be positive, thus DBH is systematically overestimated. The convex hull (CH) method defines a minimum bounding polygon for the cross section of a tree and DBH is estimated as the diameter of a circle with an area identical to the area of the bounding polygon; DBH can be underestimated if the stem is not sufficiently covered by photos from all sides. The direct least square circle (C) and least square ellipse (E) fitting techniques find the parameters of the best fitting circle and ellipse, respectively, for a given tree [30]. In contrast to the CB and CH methods, the least squares methods are not defined by bounding geometries, and therefore they are supposed to be more robust methods with noisy data compared with bounding approaches. In addition, because of their predefined forms, the least squares methods should not be affected by incomplete data of perimeters of tree cross sections. The accuracy of DBH estimation was expressed as bias (mean of errors) and root mean square error (RMSE). An N-way analysis of variance was used to test the effects of the following factors on the mean DBH error: (1) method of DBH estimation (i.e., CB, CH, C, and E), (2) type of sensor (i.e., Phantom 4 Pro and Mavic Pro), and (3) species (i.e., spruce and beech).

For a more detailed accuracy analysis, the point cloud of the spruce forest stand acquired with the DJI Phantom 4 Pro was chosen. The aim of the analysis was to verify a dependency of the accuracy of DBH estimation on the number of images containing a particular tree stem and on the number of directions from which images of a particular tree stem were taken. For each image, the area covered by the image was estimated from the position, direction, and field of view angle of the camera. If the stem cross section at 1.3 m above the ground was inside the field of view of a particular camera position and it was within the chosen effective distance limit of 15 m, the image was considered as the source image for the tree. The observation angle was calculated for each source image of a tree. Observation angles were generalized and classified into 16 observation directions. Finally, the relation between the number of source images and the number of observation directions on the error of DBH estimation was investigated with a linear regression analysis in order to determine the influence of number of observations on accuracy of resulting point cloud.

## 4. Results

The results are presented in three sections representing the main parts of the study. First, the acquired point clouds are described. Further, accuracy of point clouds is assessed through the analysis of DBH estimation and statistically evaluated, and finally, the point cloud quality is evaluated in respect to spatial properties and image coverage.

### 4.1. Imagery and Point Clouds

**Figure 3:**
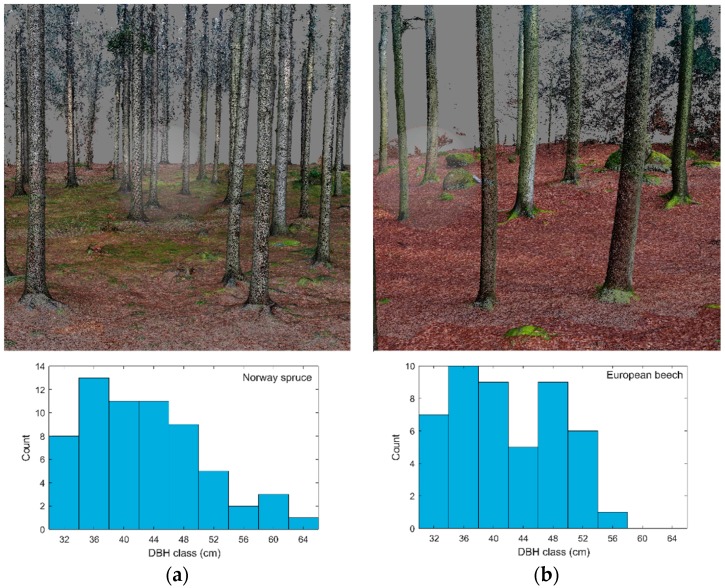
The reconstructed forest structure of the spruce (**a**) and beech (**b**) research plots. Images represent visualization of 3D point clouds, and histograms show the reconstructed distribution of DBH based on estimation from point clouds using E method.

All four image sets (two forest stands recorded by two different sensors) were successfully processed, which included image alignment and creation of dense 3D point clouds. The success rate of image alignment and numbers of points in created dense clouds are shown in Table 3. In all cases, the alignment success rate was greater than 80%. The visualization of point clouds of both research plots derived from images acquired with the Phantom 4 Pro display almost noiseless point clouds that represented the forest stand structure very well (Figure 3).

### 4.2. Diameter Estimation

The 68 and 55 trees in the spruce and beech research plots, respectively, were all identified in the point clouds. The DBH was estimated for all trees in the research plots. The results of the N-way analysis of variance indicated that the mean errors, as well as the mean absolute errors, were significantly influenced by the methods of DBH estimation and the type of sensor used. The bounding circle method showed significantly higher absolute errors than the other three methods, and the absolute error of the E method was the lowest. The CB method had the highest positive errors, with lower positive errors using the E method. Both CH and C methods showed negative errors, and their means did not differ. The same result was obtained by multiple comparisons using a paired *t*-test with Bonferroni corrections. Simultaneously, one-sample *t*-tests determined that the overall overestimation of methods CB and E and the underestimation of methods CH and C was systematic and significant. The significance of bias for individual models illustrates Table 4. The results of N-way analysis of variance also indicated that the DBH estimations based on point clouds derived from the Phantom 4 Pro images showed a significantly lower error than the estimates derived from the Mavic Pro. No difference in mean errors was found between the spruce and beech stands.

### 4.3. Spatial Distribution and Predictors of Error

**Figure 4:**
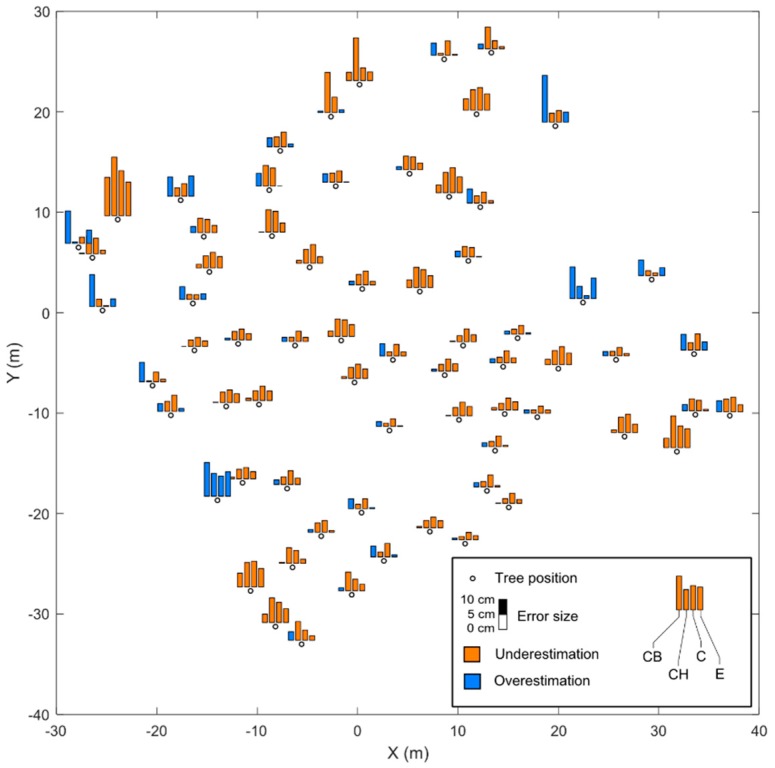
Tree positions in the spruce research plot and their respective errors of DBH estimation for all four methods from the Phantom 4 Pro data.

Figure 4 shows tree positions in the spruce research plot and their respective errors of DBH estimation for all four methods. The highest errors were distributed near the edges of the research plot, where image coverage was lower compared to the center; the result was that stems around the plot edges were not completely modelled from all sides, thus causing underestimation of DBH, especially with the CH method, or contain more pronounced noise causing overestimation of DBH, particularly for the CB method.

**Figure 5:**
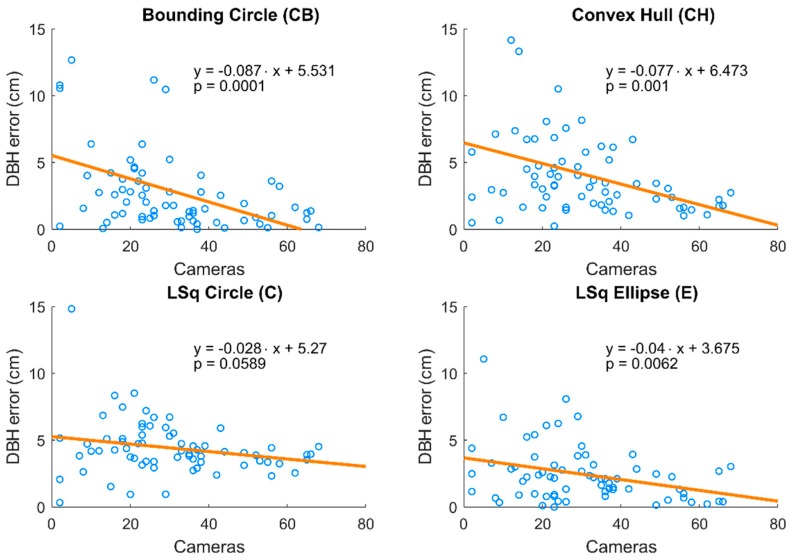
Relationships between the number of observation cameras and DBH estimation error for four methods of DBH estimation. Regression line in each chart demonstrates the decreasing trend of error size with increasing amount of observation cameras. Equations of regression lines and *p*-values for slope parameters are provided.

The estimated number of observing cameras for each tree varied between 2 and 68, and trees in the inner part of the research plot were observed more frequently, while trees at the edges were less frequently observed. As indicated in Figure 5, there was a significant dependency of the absolute DBH error on the number of images that recorded the tree. A decline of absolute error with an increasing number of observing images was found for all methods of DBH estimation, significant in all cases with the exception of the least squares circle fitting method. This result is in accordance with findings of previous studies [17] reporting linear decline of position error of photogrammetric points with an increasing number of observing cameras.

**Figure 6:**
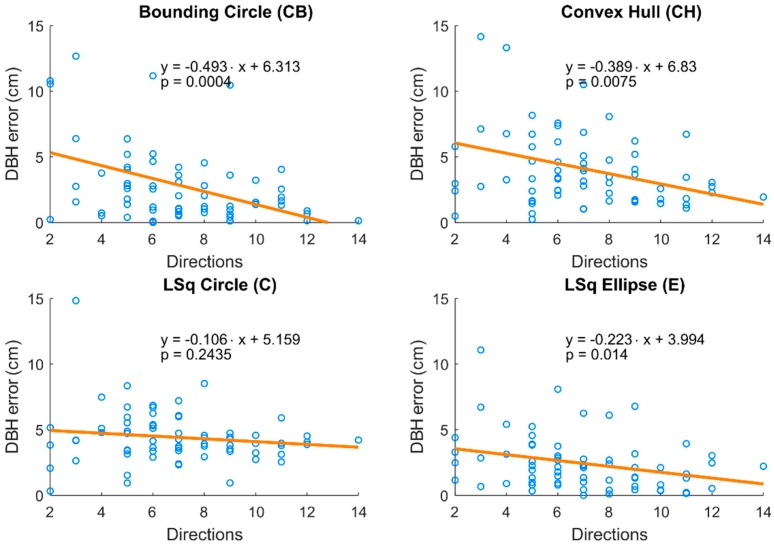
Relationships between the number of observation directions and DBH estimation error for four methods of DBH estimation. Regression line in each chart shows the decrease in error with increasing number of the observation directions. Equations of regression lines and *p*-values for slope parameters are provided.

No tree in the research plot was covered by photos from all 16 directions; the lowest recorded number of observing directions was two, and the highest was 14 of 16 directions. Much like the number of cameras, the number of observing angles influenced the accuracy of DBH estimation. Again, methods BC, CH, and E showed a significant decline of absolute DBH error with increasing number of observing angles (Figure 6).

## 5. Discussion

Our study demonstrated the ability of two UASs, the Phantom 4 Pro and the Mavic Pro, to successfully perform a highly demanding flight in two forest stands with little or no support of GNSS using inside flight capabilities. The UASs collected imagery suitable for developing an accurate 3D photogrammetric point cloud using multi-view stereophotogrammetry techniques.

A similar result—a 3D point cloud describing the structure of the forest stand—can be achieved using similar methods of terrestrial photogrammetry with a hand held digital camera [2,26]. However, the elevated position of the camera when carried by a UAS allows for the reconstruction of a considerably higher portion of the stem profile, depending on the flight height over the surface. Our study used a flight height of 8 m, which allowed us to reconstruct the stem surfaces up to a height of 10 m. Diameters derived from higher portions of the stem allows us to predict the stem curve based on diameter measurements from lower on the stem [31,32]. With the stem curve known, further valuable data can be derived, such as the volume of any specified logs and the assortment structure can be estimated. Contrary to some of the other mentioned photogrammetry studies, our approach did not require any ground control points for scaling and orienting the point clouds, which considerably accelerates and simplifies the process and it represents a distinct shift towards utilizing UASs being considered as a practical method for data acquisition.

It must be stated that the presented method would not be applicable in all types of forests. The main limitation is the requirement of open space for the flight, which will typically limit them to even-aged forest stands with sufficient distance between tree stems (a minimum of 2 m), higher tree crown bases, and the absence of high understory vegetation or individual branches in the flight space. The presence of thin dry branches may be particularly limiting because such small objects are undetectable by the obstacle avoidance sensors of the UASs. However, precise inventory investigations would typically be performed in mature, high-quality stands that have been actively managed, and such stands generally match the requirements.

The tested DBH estimation methods produced results comparable to other studies that used terrestrial photogrammetry (TP), terrestrial laser scanning (TLS), and aerial laser scanning (ALS) from UAS platforms in previous studies, as presented in Table 5. The ALS error applies only to the successfully detected trees—not all stems can be detected due to limited penetration of laser beams through tree canopies; [7] reported a 76.6% success rate for tree detection using ALS. Aerial laser scanning from airplanes and aerial photogrammetry are not mentioned in the comparison because they do not allow direct derivation of individual DBHs from the data. Rough estimates of equipment costs are USD $500 for TP, $100,000 for TLS, and $300,000 for ALS. The price of UASs used in this study was approximately USD $1500 and $1000 for the DJI Phantom 4 Pro and DJI Mavic Pro, respectively.

For this study, we used only simple diameter estimators, which produced some mixed results. Some of the DBH estimation methods led to the underestimation of DBH, while others to systematically produced overestimates, which indicates that the errors were caused by the DBH estimation methods rather than the method of point cloud derivation. Therefore, more advanced fitting methods may significantly improve DBH estimations. It is important to note also that the relationship between mean error (bias) and root mean square error; the latter is influenced by extreme values, while the former expresses the extent of systematic error. The DBH errors showed small bias and large RMSE, particularly for the Mavic Pro, which means that errors of overestimation and underestimation were equal. The lower camera resolution of Mavic Pro may limit feature detection in images, thus causing inaccurate image alignments or poor point cloud derivations in areas with lower coverage of images.

The number of observing cameras and the directions were determined by the selected effective distance limit, which was set to 15 m for this study. This is a reasonable estimation of the average distance that allows for the observation of tree stems with sufficient detail and it is less likely that the view will not be obstructed by nearby trees. However, although the chosen value influences the counts of observed images and directions, it does not change the decreasing trend of error with higher image coverage. Obviously, the number of observation images correlates with the number of observation directions, but each of the predictors implicates a different aspect of the image coverage. The number of images positively affects the accuracy of derived 3D point positions [17], but the perimeters of stem cross sections can be correctly modelled to help provide DBH estimates only if the stem is photographed from multiple directions.

## 6. Conclusions

We reported on the possible use of small commercial unmanned aerial systems (UASs) equipped with a high-precision vision positioning system and image geotagging capability, namely the DJI Phantom 4 Pro and the DJI Mavic Pro, for 3D forest structure mapping within forest stand interiors with the use of multi-view sterephotogrammetry techniques. Images of two research plots of mature single-species stands of Norway spruce and European beech were successfully acquired during manually guided flights of the forest stand interiors with the support of obstacle sensing sensors and avoidance systems of the UASs. Acquired image sets were used for subsequent derivation of georeferenced 3D point clouds that described the structure of the forest stand and allowed for reasonable estimates of forest stand parameters, such as tree positions, diameters, or stem curves.

Comparison of the UASs showed that the images acquired with the DJI Phantom 4 Pro had better quality and a greater level of detail than the images from the DJI Mavic Pro; this was a result of its larger sensor and higher sensor resolution. Consequently, the point cloud derived from Phantom 4 Pro images provided more accurate estimates of DBH, with a mean error (bias) of −1.17 cm and 0.27 cm in spruce and beech stands, respectively, using the least squares ellipse fitting method. It was demonstrated that the DBH estimation error declines with an increasing number of observation images and observation directions. The highest error was distributed around the edges of the research plots where the image coverage was lower compared to the interior portions of the plots.

## Figures and Tables

**Table 1:** Research plot parameters.

Research Plot	Spruce	Beech
Species composition percentages (# trees)	97% Norway spruce (67) 3% European beech (1)	100% European beech (55)
Age (years)	102	110
Mean diameter (cm)	43.7	41.8
Mean height (m)	33.4	31.2
Crown base height (m)	14	12
Stocking	90%	100%
Tree density (trees/ha)	290	270
Volume (m^3^/ha)	560	540

**Table 2:** Key features of the two sensors used in the study.

Sensor	DJI Phantom 4 Pro	DJI Mavic Pro
Sensor size	1 inch	1/2.3 inch
Sensor resolution	20 MPix	12 MPix
Max. ISO	12,800	3200
Aperture	f/2.8–f/11	f/2.2 fixed
Shutter	Mechanical	Electronic

**Table 3:** Characteristics of collected data, including: counts of captured and aligned images, numbers of tie points used for aligning images, and numbers of points in the resulting dense 3D point clouds.

Research Plot	UAS	Imgs. Captured	Imgs. Aligned	Tie Points	Points
Spruce	Phantom 4 Pro	1099	895 (81%)	327 thous.	29 mil.
	Mavic Pro	461	416 (90%)	998 thous.	10 mil.
Beech	Phantom 4 Pro	581	531 (91%)	502 thous.	22 mil.
	Mavic Pro	767	697 (91%)	185 thous.	17 mil.

**Table 4:** Bias and root mean square error (RMSE) of DBH estimations in spruce (upper part) and beech (lower part) research plots for the following methods: bounding circle (CB), convex hull (CH), least squares circle (C), and least squares ellipse (E). Asterisks represent statistically significant difference from zero bias.

	Phantom 4 Pro				Mavic Pro			
	Bias (cm)	Bias (%)	RMSE (cm)	RMSE (%)	Bias (cm)	Bias (%)	RMSE (cm)	RMSE (%)
CB	1.55 *	2.89	4.34	9.66	11.29 *	27.76	20.39	54.48
CH	−3.77 *	−9.09	5.31	13.3	−2.18	−3.76	11.79	30.91
C	−4.19 *	−10.11	4.87	12.41	−1.98	−3.19	9.67	23.79
E	−1.17 *	−3.14	3.21	8.18	5.47	14.57	18.66	49.89
CB	3.04 *	7.84	4.5	11.95	8.84 *	22.44	11.92	31.96
CH	−2.64 *	−6.26	4.1	9.95	0.06	0.2	5.46	13.82
C	−3.37 *	−8.31	3.63	9.18	−0.4	−0.97	5.48	15.92
E	0.27	0.69	2.63	7.01	6.16 *	15.69	9.71	26.48

**Table 5:** Comparison of DBH estimates with accuracies of DBH estimates from 3D point clouds acquired with terrestrial photogrammetry (TP), and terrestrial laser scanning (TLS), and UAS borne aerial laser scanning (ALS) from other studies. The results displayed from our study were estimated using the E method and Phantom 4 Pro UAS.

Method	DBH Error (cm) (RMSE if not Mentioned Otherwise)	Study
TP	0.9–1.19	[2]
	2.8–9.5	[33]
	4.41–5.98	[26]
TLS	3.2–4.2	[34]
	1.11–3.73	[35]
	1.7 ± 2.8 (mean ± SD)	[36]
	−0.96–0.93 ± 1.23–2.47 (mean ± SD)	[37]
	0.90–1.90	[38]
	0.7–7.0	[4]
ALS	0.8–4.7 (median)	[7]
	4.24	[3]
Our approach	2.63–3.21	
